# Giant lipoma: an unusual cause of carpal tunnel syndrome

**DOI:** 10.4314/pamj.v9i1.71205

**Published:** 2011-07-18

**Authors:** Divesh Jalan, Bhavuk Garg, Kanniraj Marimuthu, Prakash Kotwal

**Affiliations:** 1 All India Institute of Medical Sciences, Ansari Nagar, New Delhi, India

**Keywords:** Lipoma, carpal tunnel syndrome, median nerve, nerve compression

## Abstract

Carpal tunnel syndrome, in its idiopathic form, is an extremely common entrapment neuropathy in the clinical practice however secondary compressive causes are rare. Among secondary causes, tumors are even rarer. Although lipomas are the most common soft tissue tumor in the body, <5% of the benign tumors of the hand are lipomas. A 48-year old manual laborer man presented to us with a two-year history of numbness, tingling and burning pain in the palmar surface of the left hand and fingers along with a progressively increasing swelling in the hand and wrist. His medical history was unremarkable and no trauma episode was reported. According to the clinical examination and the result of median nerve conduction study (NCS) the diagnosis of carpal tunnel syndrome was established. Operative release of the transverse carpal ligament was subsequently performed along with excision of the lipoma using extensile open approach. Intraoperatively, median nerve and its digital branches were found to be stretched over the giant lipoma causing substantial compression to median nerve. Histopathological findings of the resected mass were consistent with lipoma. After two years the patient was pain-free without any sign of tumor recurrence. Lipomas are infrequently seen in hand and wrist, however giant lipoma as a cause of secondary carpal tunnel syndrome is even more rare, which makes this case interesting.

## Introduction

Carpal tunnel syndrome, in its idiopathic form, is an extremely common entrapment neuropathy in the clinical practice; however secondary compressive causes are rare. Among secondary causes, tumors are even rarer. There are two broad categories of tumors that can compress a peripheral nerve including the median nerve at the carpal tunnel: tumors with a neural sheath origin and those that have a non-neural sheath origin. Lipomas are the most common soft tissue tumor of non-neural origin in the body however they account for less than 5% of benign tumors of the hand. There are reports of lipofibromatous hamartoma of the median nerve [1], flexor tendon sheath lipoma [2], even occult palmar lipoma [3] causing carpal tunnel syndrome but a giant lipoma, because of its rarity in hand, has not been reported in the English literature to the best of our knowledge. We describe a rare case of secondary carpal tunnel syndrome due to a giant lipoma compressing the median nerve and its digital branches.

## Case report

A 48-year old manual laborer, right-hand-dominant presented to us with a two-year history of numbness and tingling sensation in the palmar surface of left hand and radial three fingers along with a progressively increasing swelling in the left wrist and hand. During the last six months the patient reported burning pain in the same area – causing him to wake up frequently during the night time hours – and progressive inability to perform his regular occupation. No episode of trauma or other medical problems were mentioned.

**Figure 1:**
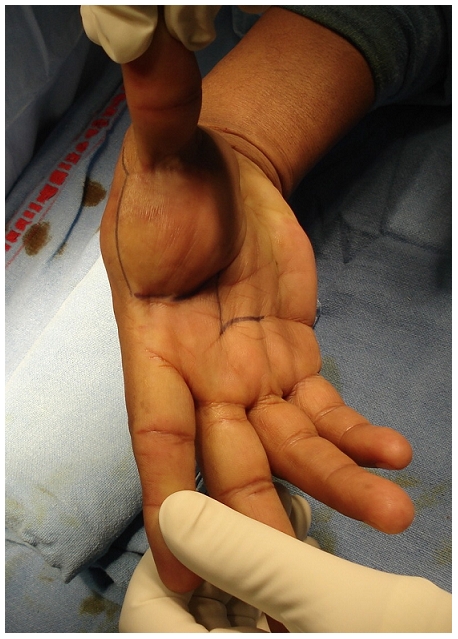
Photograph of the lesion showing the incision marking

On clinical examination there was a firm swelling in the palmar aspect of left hand and wrist measuring 8cm x 4cm x 3cm (Figure 1). It was non tender, relatively adherent swelling with well defined margins. There was a sensory loss in the area innervated from the median nerve as compared with the contralateral hand and Tinel′s sign was positive at the wrist. The patient had decreased grip strength; the phalen manoeuver was also positive.

**Figure 2:**
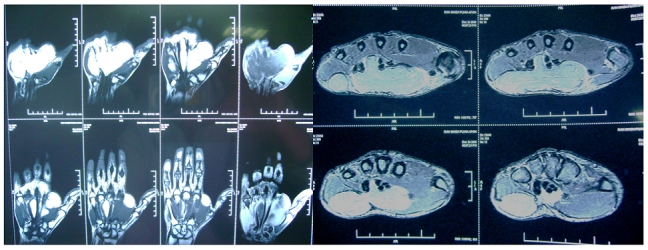
MRI showing actual extent of tumor (bright signal on T1 images -transverse and coronal sections)

X rays of the wrist and hand including carpal tunnel view showed increased soft tissue shadow with no bony abnormality. Electophysiological study confirmed the diagnosis of carpal tunnel syndrome. MRI study confirmed the diagnosis of a benign tumor, possibly Lipoma, showing bright signal on T1 images and dark signals on fat suppressed images (Figure 2).

**Figure 3:**
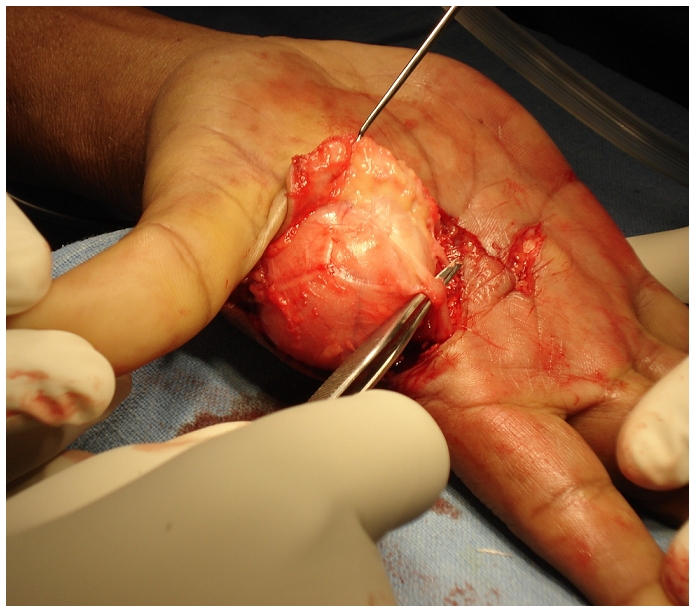
giant lipoma stretching the median nerve and its branches

Through an extensible palmar approach, transverse carpal ligament was incised. The median nerve and its branches were found stretched over a large swelling thereby compressing the nerve (Figure 3).

**Figure 4:**
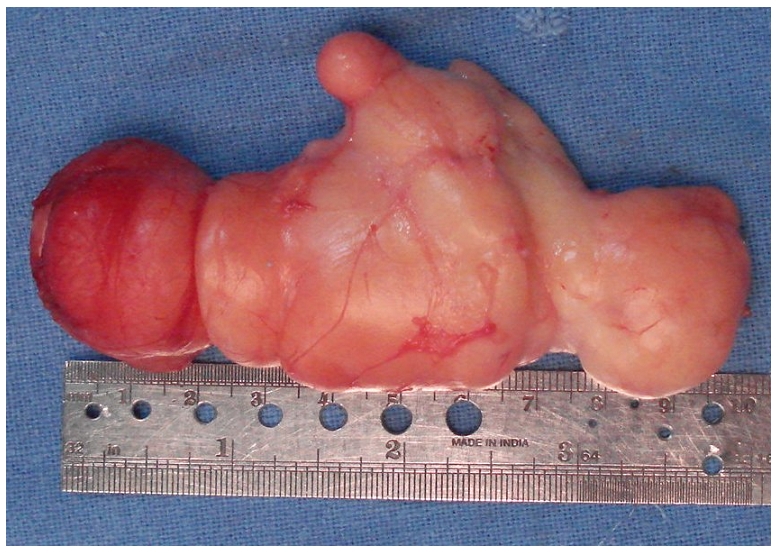
resected giant lipoma measuring about 10x5x3cm

The tumor was arising in mid palmar space extending in the hand and wrist with no connection suggesting its origin. The mass was carefully dissected from its neighbouring structures and shelled out in toto. The mass measured 10 x 5 x 3cm (Figure 4). It was lobulated, yellow to brown in colour with sparse vascularity. Macroscopically the diagnosis of lipoma was obvious. The resected specimen was sent for histopathological examination which confirmed the diagnosis of lipoma.

**Figure 5:**
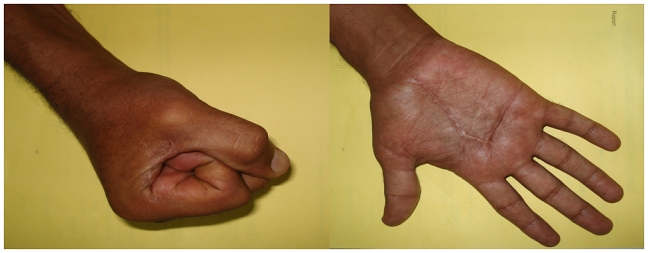
At 2 years follow up, no recurrence with good hand grip

At 2 years follow up, the patient showed significant clinical improvement with no pain and mild improvement in sensory function. His hand grip strength also improved and he returned to his previous occupation. There was no recurrence of the mass at 2 year follow up (Figure 5).

## Discussion

Carpal Tunnel Syndrome is known to be the most common compressive neuropathy in the hand. Although most cases of Carpal Tunnel Syndrome are idiopathic, the rare identifiable causes could be the space occupying lesions such as the swelling of tendon sheath, distal radius fracture, rheumatoid arthritis, volar dislocation of lunate, ganglion and gout etc. If these space occupying lesions are not discovered or misdiagnosed and treated as idiopathic Carpal tunnel syndrome, the patient's symptoms will not improve.

Idiopathic Carpal tunnel syndrome is generally known to occur bilaterally. According to Bagatur et al.[4], 66% of patients with unilateral Carpal tunnel syndrome symptoms exhibit abnormal results in nerve conduction tests on contralateral hand. Therefore, when patient's symptoms and nerve conduction test show unilateral abnormalities, it is necessary to suspect other reasons for Carpal tunnel syndrome besides idiopathic Carpal tunnel syndrome. Nakamichi et al.[5] reported that out of 20 patients with abnormal unilateral symptoms and unilateral positive electrophysiologic tests, besides 7 (35%) idiopathic adults, the rest of the patients (65%) showed various causes for median nerve compression, such as occult ganglion, occult calcified mass, tuberculous synovitis, nonspecific synovitis etc.

In order to rule out space occupying lesions in unilateral Carpal tunnel syndrome patients who exhibit swelling or palpable mass in the volar wrist area during physical examination or patients who exhibit radio-opaque lesion discovered during plain X-ray, further special studies (ultrasonogram, CT, MRI) should be performed [6, 7].

Mason classified lipomas of the hand into those lying within (endovaginal) or outside (epivaginal) of tendon sheath [8]. Depending on their anatomic location with the parent nerve, four major types of lipomatous masses can be identified in the extremities: soft tissue lipoma, intraneural lipoma, lipofibromatous hamartoma, and macrodystrophia lipomatosa.

Soft tissue lipomas are true benign neoplasms originating from adipose cells. They are generally well-defined masses occurring in the subcutaneous, subfascial, and intermuscular planes. Subcutaneous lipomas generally form soft, lobulated masses causing no symptoms except for appearance. Deep lipomas located in the subfascial or intermuscular planes may cause extrinsic compression of major nerve trunks. Deep lipomas lie beneath the palmar fascia and are not easily palpable. Lipomas in the deep subfascial planes of the palm may functionally impair the hand, either by mechanical restriction from the sheer mass or by extrinsic compression of the median or ulnar nerves, as in this case.

Recently, endoscopic or minimal invasive surgery has been preferred for carpal tunnel syndrome. However, in cases such as this one involving space occupying lesions, symptoms do not improve unless open transverse carpal ligament release is performed in conjunction with removal of the space occupying lesion.

The use of MRI scans is an excellent way to examine soft tissue, and also has the advantage to make it possible to diagnose the exact location and border of lesion and also the involvement of surrounding tissue.

## Conclusion

The diagnosis of soft tissue tumor causing carpal tunnel syndrome requires a high index of suspicion, particularly in unilateral cases. A detailed preoperative planning including MRI is essential for successful outcome. Presence of such secondary causes warrants the use of extensile open approach and is a contraindication for endoscopic procedures.
